# Barriers to Creating Scalable Business Models for Digital Health Innovation in Public Systems: Qualitative Case Study

**DOI:** 10.2196/20579

**Published:** 2020-12-10

**Authors:** Leah Taylor Kelley, Jamie Fujioka, Kyle Liang, Madeline Cooper, Trevor Jamieson, Laura Desveaux

**Affiliations:** 1 Institute for Health System Solutions and Virtual Care Women's College Hospital Toronto, ON Canada; 2 Unity Health Toronto, ON Canada; 3 Institute of Health Policy, Management and Evaluation University of Toronto Toronto, ON Canada

**Keywords:** digital technologies, telemedicine, innovation diffusion, health policy, evaluation study, reimbursement, incentive, mobile phone

## Abstract

**Background:**

Health systems are increasingly looking toward the private sector to provide digital solutions to address health care demands. Innovation in digital health is largely driven by small- and medium-sized enterprises (SMEs), yet these companies experience significant barriers to entry, especially in public health systems. Complex and fragmented care models, alongside a myriad of relevant stakeholders (eg, purchasers, providers, and producers of health care products), make developing value propositions for digital solutions highly challenging.

**Objective:**

This study aims to identify areas for health system improvement to promote the integration of innovative digital health technologies developed by SMEs.

**Methods:**

This paper qualitatively analyzes a series of case studies to identify health system barriers faced by SMEs developing digital health technologies in Canada and proposed solutions to encourage a more innovative ecosystem. The Women’s College Hospital Institute for Health System Solutions and Virtual Care established a consultation program for SMEs to help them increase their innovation capacity and take their ideas to market. The consultation involved the SME filling out an onboarding form and review of this information by an expert advisory committee using guided considerations, leading to a recommendation report provided to the SME. This paper reports on the characteristics of 25 SMEs who completed the program and qualitatively analyzed their recommendation reports to identify common barriers to digital health innovation.

**Results:**

A total of 2 central themes were identified, each with 3 subthemes. First, a common barrier to system integration was the lack of formal evaluation, with SMEs having limited resources and opportunities to conduct such an evaluation. Second, the health system’s current structure does not create incentives for clinicians to use digital technologies, which threatens the sustainability of SMEs’ business models. SMEs faced significant challenges in engaging users and payers from the public system due to perverse economic incentives. Physicians are compensated by in-person visits, which actively works against the goals of many digital health solutions of keeping patients out of clinics and hospitals.

**Conclusions:**

There is a significant disconnect between the economic incentives that drive clinical behaviors and the use of digital technologies that would benefit patients’ well-being. To encourage the use of digital health technologies, publicly funded health systems need to dedicate funding for the evaluation of digital solutions and streamlined pathways for clinical integration.

## Introduction

### Background

Digital technology offers the potential to efficiently meet the health care demands of a population growing in both size and complexity, without sacrificing quality. Administrators and health organizations are increasingly seeking technologies from the private health care market to achieve this aim [1]. Despite these efforts and accompanying investments [2], health systems struggle to translate innovation into clinical practice [3,4]. Failure to clearly define the health care marketplace and the parameters for entry present considerable challenges for private sector small- and medium-sized enterprises (SMEs) [5]. Maximizing value in public-private partnerships for digital health requires motivating the development of innovative solutions by the private industry, encouraging integration of those solutions into clinical spaces, and assuring ongoing refinement of the tools and surrounding clinical models [6].

### Driving Innovation in Public Health Systems

Creating an environment in which emerging solutions meet the needs of a public health system requires that these private entities develop sustainable business models within that system [7]. However, health systems are characterized by complex and fragmented care models, alongside a myriad of regulatory models and incentive structures that are often incongruent with private sector business models [7]. Further, the ability to demonstrate value to public systems is challenging due to complex clinical practices, organizational processes, and provider workflows [8]. The issue of incentives and payment models to support the use of digital tools that promote patients’ well-being cannot be solved through accelerators and academic medical centers. If health systems want to increase the use of digital tools that can reduce the use of health services and alleviate the burden on acute care facilities, there is a need for incentive models that support their adoption. Currently, digital models that rely on clinicians monitoring data are affected because this model has no economic incentive to encourage the clinician to participate and incurs a fear of liability [9]. Complex and often conflicting actors in health care (eg, purchasers, providers, and producers of health care products) make developing value propositions associated with those products highly challenging [10]. Electronic health records are a telling precedent, as health system payers implemented them to solve issues primarily for the payers, including data collection for administrators and billing, at times at the expense of clinician experience. This has led to feelings of frustration and burnout associated with their use [11]. Policy makers need to consider incentive models for innovators to develop products that support direct clinical needs *and* help solve system problems. There is a recognized need for health systems to align reimbursement, policies, and infrastructure with the unique care pathways involving digital health solutions to increase their uptake [12,13].

Innovation in digital health is largely driven by SMEs, which include businesses with fewer than 500 employees. SMEs develop digital technologies to solve clinical and administrative problems, with a view of selling them to health organizations, public and private health system payers, and directly to consumers in Canada and internationally. SMEs face unique challenges compared with larger companies in the field because of their lack of connections and leverage in the system and limited resources and access to data to test and develop their solutions. To overcome these barriers, SMEs must develop an efficient clinical model (to promote provider buy-in and value) [14], a strong business model (to promote financial sustainability) [15], and a reliable method to generate evidence (to promote adoption of safe, effective, and valuable technologies of interest to a public payer) [16]. Insights from real-world SMEs attempting to create sustainable and scalable enterprises within the constraints of a public system (rather than research-generated technologies) are critical to creating more symbiotic partnerships between the public system and private industry.

### Funding and Regulating Digital Health Technologies in Canada

Funding in the Canadian health system is largely determined by the Canadian Constitution, which allocates responsibility for health care delivery to the provinces. The *Canada Health Act (CHA)* guides funding allocation to provinces for health care delivery, which is collected through a federal tax [17]. The system provides a broad range of health services, divided into 3 layers: those entirely publicly funded (eg, hospitals, physicians, diagnostics), those funded through mixed public and private insurance (eg, prescription drugs, home care, mental health care), and those funded entirely through private insurance (eg, private physiotherapy, dentistry) [18]. There is some variation between provinces in services reimbursed through public insurance: the *CHA* requires that provinces cover services that are *medically necessary*; however, it is left to the provinces to interpret which services fall under this definition. Generally, services provided by a physician are considered *medically necessary*. Private insurance, often provided through employers, fills gaps in health services not covered through public insurance.

There are no regulatory requirements that cover all digital health technologies in Canada. Those meeting the definition of *medical devices*, generally those that are used for diagnosis and treatment, must be specifically approved through Health Canada; however, precise rules and guidelines are often challenging to apply [19]. Health Canada is currently exploring regulatory processes for artificial intelligence–based software that supports clinical decision making [20], which may look similar to the United States Food and Drug Administration’s precertification program for digital health. However, many of the tools created by SMEs are either not medical devices or are lower class medical devices that do not require much oversight. This reality leaves hospitals and clinics that wish to adopt digital technologies liable for their safety, efficacy, and security—before any evaluation that has proven such safety and efficacy, as all other institutions are in the same situation. This paper analyzes a series of case studies to identify current health system barriers faced by SMEs developing digital health technologies in Canada and proposes solutions to encourage a more innovative ecosystem.

## Methods

### The Market Entry Consulting Program

This paper reports on a retrospective evaluation of a real-world program, not a prospective research study with targeted recruitment strategies and protocols geared toward answering a specific question. The reports that were reviewed and qualitatively assessed to produce the content of this study were consolidated by experts in health policy and digital technology, not researchers. The Women’s College Hospital Institute for Health System Solutions and Virtual Care (WIHV) established a consultation process for SMEs to help them increase their innovation capacity and take their ideas to market, with support from the National Research Council Industrial Research Assistance Program. WIHV provided market entry consulting services to SMEs in the digital technology sector in the form of a structured assessment. The process engaged an advisory committee comprising expert advisors in key content domains (informatics, engineering, policy, funding models, and business) alongside health care providers (predominantly physicians but occasionally nurses, pharmacists, and allied health professionals) with relevant clinical expertise. Each SME had between 5 and 8 advisors review its product and business model, which varied depending on the area of practice and availability of the advisors. The advisors reviewed the SME’s business model and its product based on the features of the technology itself, the feasibility of its implementation in relevant clinical environments, the potential impact on patients and health systems, and the potential for scale and spread (Table 1). The program was promoted through WIHV’s existing network and through scientific conferences and digital technology events.

**Table 1:** Considerations to guide the evaluation of the small- and medium-sized enterprise product, clinical model, and business model.

Domain		Sample considerations
**Technology**		
	Idea	Mechanism, earlier evidence or validation, innovativeness, and problem definition
	Regulatory requirements	Safety and privacy mitigations
**Feasibility**		
	Clinician	Workflow requirements, integration, and behavioral changes
	Patient	Required engagement and education
	Institutions	Cost, training, human resource requirements, and risk
	Health systems	Cost, policy requirements, and risk
**Impact**		
	Patient	Health outcomes, experience, and quality of care
	Health systems	Cost-effectiveness and population health impact
**Scale and spread**		
	Political and economic alignment	General interest or need for a product and stakeholder alignment
	Innovator	Commitment, experience, skills, and goals
	Procurement strategy	Marketing and potential revenue

The consultation process began when an SME completed an onboarding form that provided the advisors with details on their product, marketing strategy, perceived benefit and burden on the health system, expertise, customers, end users, and privacy and security considerations (Multimedia Appendix 1). SMEs identified barriers they had faced in introducing their product to the health system that they wanted the advisors to address. The onboarding form was refined from its initial iteration to efficiently extract relevant information from clients to inform the analysis. SMEs were required to answer all questions. All advisors then independently reviewed the SME’s responses following a list of prompts (Table 1). The onboarding and assessment framework for the market entry consulting program was developed using an iterative, co-design approach with stakeholders and experts from multiple disciplines (including medicine, business, technology, health services, and innovation) to capture a broad range of factors important to the success of digital health companies. It was refined based on the experiences and recommendations of the advisors throughout the program.

On the basis of the information provided by SMEs in the onboarding form (including information about the clinical and business models and any explicit challenges noted), the advisors identified system barriers that would make entry into the health system challenging. Each member provided insights and recommendations relative to their expertise to support the SME in navigating the complexities of the health system. Depending on the clinical area of the technology developed by the SME, a content expert (eg, a pharmacist) may be engaged in the review. Insights, barriers, and recommendations were then consolidated to produce a single report that outlines key formative recommendations for the SMEs, which was reviewed by the advisors for accuracy and final comment. A draft of the report was then shared with the SME, after which a debrief meeting was held with all parties to review and refine recommendations. The report was then revised based on discussion, and the final report was sent to the SME (Textbox 1).

**Steps in the market entry consulting process.**

The market entry consulting program reported on in this paper involves a multi-step engagement process with small- and medium-sized enterprises (SMEs), as outlined here:

1. Women’s College Hospital Institute for Health System Solutions and Virtual Care (WIHV) had the initial contact with SME.
2. The SME provided the completed onboarding form.
3. Committee members reviewed the SME onboarding form.
4. Committee members provided insights and recommendations.
5. WIHV consolidated recommendations into a draft report.
6. The committee reviewed the draft report.
7. The committee met with the SME to review recommendations and insights.
8. WIHV revised the report and provided it to the client.

### Ethics

This initiative was formally reviewed and approved by the Chair of the Research Ethics Board for program evaluation projects at Women’s College Hospital (Research Ethics Board #2017-0127-E).

### Data Collection and Analysis

Data included onboarding forms and final recommendation reports produced for companies assessed by the program from 2016 to 2018. SME data were extracted from the onboarding form to describe the SMEs’ characteristics, including the clinical area where a tool would be implemented (eg, cardiology, wound care), primary function (classified according to the National Health Service Evaluation Framework [21], eg, active monitoring), description of the tool and service, intended users (eg, patients, physicians), intended payers (eg, hospitals, physicians), payment model (eg, pay per use), data collected by the tool/service (eg, Personal Health Information), data source (eg, wearable, patient reported), and studies conducted (ie, self-study and/or external evaluation).

We conducted an inductive thematic analysis across all SME reports to identify common health system barriers experienced by SMEs in a digital health innovation who completed the market entry consulting program, as described in the reports produced by the market entry consulting program advisors. Two members of the research team (JF and KL) independently coded the first 3 reports using NVivo version 11 (QSR International). JF and KL met with a third member of the research team (LK) to review the initial codes and resolve discrepancies to develop a preliminary coding structure. JF and KL then applied and expanded upon the resulting coding structure in the remaining reports. Data were stored and organized into emergent themes in NVivo. Final themes were identified through team discussion to identify the overarching, pervasive barriers to digital technology innovation faced by several innovators.

## Results

### Characteristics of SME Products

Data from 25 SMEs were reviewed. Table 2 provides an overview of companies’ functions, users, payment model, data collected, and evaluations conducted. A total of 11 technologies developed by the participant SMEs coordinated or optimized administrative functions in public or private health institutions (eg, a tool to improve patient transitions). Moreover, 7 technologies actively monitored chronic conditions by collecting and sending patient-generated data to health care providers for review either in real time or intermittently. The remaining 7 technologies had a variety of purposes, including disease self-management, advanced diagnostics, or clinical decision support.

**Table 2:** Characteristics of small- and medium-sized enterprises’ clinical model, business model, and their digital health tools as extracted from the onboarding forms completed by the small- and medium-sized enterprises, sorted by Clinical area.

Clinical area and primary function			Description of the technology/service	Intended users	Intended payers	Payment model	Data collected by the tool/service	Data source	Studies conducted
**Geriatrics, cardiology, and respirology**									
	**Active monitoring**								
		SME^a^ 1	Active monitoring of vitals for high-risk patients	Patients, physicians, and pharmacists	Patients, public institutions (hospitals and clinics), and physicians	Pay per use	Collects PHI^b^	Wearable	Self-study
**Cardiology**									
	**Active monitoring**								
		SME 2	Active monitoring of vitals for high-risk patients	Patients	Public institutions (hospitals and specialist clinics) and private insurance	One-time purchase, rental fee per user	Collects PHI	Wearable	External evaluation and self-study
**Cardiology and respirology**									
	**Active monitoring**								
		SME 3	Active monitoring of vitals for high-risk patients	Physicians, patients, and researchers	Public institutions (hospitals and clinics), private insurance, and contract research organizations	One-time purchase	Collects PHI	Wearable	External evaluation and self-study
**Dermatology**									
	**Active monitoring**								
		SME 4	Active monitoring of dermatology	Physicians and nurses	Public institutions (hospitals and clinics) and public/private institutions (home care)	Subscription fee per user	Collects PHI	Images taken by provider	Self-study
		SME 5	Active monitoring of dermatology	Physicians and nurses	Public institutions (hospitals and clinics) and public/private institutions (home care)	One-time purchase, subscription fee	Collects PHI	Images taken by patient	External evaluation and self-study
		SME 6	Active monitoring of dermatology	Patients and health care providers	Public/private institutions	One-time purchase, subscription fee	Collects PHI	Patient reported	External evaluation and self-study
**Mental health**									
	**Active monitoring**								
		SME 7	Active monitoring of mental health treatment	Therapists and patients	Public institutions (hospitals and primary care clinics), private health institutions (psychotherapy clinics), and private insurance	Subscription fee per user	Collects PHI	Patient reported	Self-study
	**System services**								
		SME 23	Coordination of web-based therapy appointments	Patients	Patients and private insurance	Pay per use	Collects PHI	Patient reported	Self-study
**Chronic disease**									
	**Self-manage**								
		SME 8	Self-management of chronic disease	Patients and researchers	Private insurance and contract research organizations	Subscription fee per user	Collects PHI	Patient reported	External evaluation and self-study
		SME 9	Self-management of chronic disease	Patients	Patients	Subscription fee	Collects PHI	Wearable	External evaluation and self-study
		SME 10	Monitoring and managing chronic disease using wearables	Patients	Patients and private insurance	Subscription fee per user	Collects PHI	Wearable	External evaluation and self-study
	**Diagnose**								
		SME 12	Diagnosis and monitoring of chronic disease	Physicians, nurses, caregivers, and research teams	Patients	Subscription fee per user	Collects PHI	Patient reported	Self-study
**Nonspecific**									
	**Calculate**								
		SME 11	Artificial intelligence–based insights into disease patterns	Physicians, nurses, and research teams	Private insurance and contract research organizations	Subscription fee per user	No PHI, de-identified data	Large clinical data sets	Self-study
	**System services**								
		SME 17	Administrative and clinical workflow optimization	Health care providers and administrators	Public institutions (hospitals and chronic disease agencies) and private insurance	Subscription fee	Collects PHI	EMR^c^	External evaluation and self-study
		SME 18	Administrative optimization of clinics	Administrators and patients	Private institutions (pharmacies) and public institutions (primary care clinics)	Revenue-share model	Transmits PHI (does not store or collect)	Patient reported	Self-study
		SME 24	Capture previsit patient information/send information to optimize clinical workflow	Patients, health care providers, and administrators	Other vendors (white label) and public institutions (hospitals and clinics)	Pay per use	Collects PHI	EMR	Self-study
		SME 25	Capture previsit patient information/send information to optimize clinical workflow	Patients, physicians, and administrators	Public institutions (hospitals and clinics)	Subscription fee per user	Collects PHI, patient experience survey	EMR, patient reported	External research and self-study
**Acute disease**									
	**Diagnose**								
		SME 13	Diagnosis of certain acute diseases	Patients	Patients and private insurance	One-time purchase	No PHI	N/A^d^	External evaluation
**Postacute care**									
	**Inform**								
		SME 14	Postacute care discharge planning	Patients and caregivers	Public/private institutions (home care) and public institutions (hospitals)	Pay per use	No PHI, patient surveys	Patient reported	Self-study
**Pharmacy**									
	**System services**								
		SME 15	Medication management and renewal	Pharmacists, physicians, and patients	Private institutions (pharmacies) and private insurance	Pay per use	Collects PHI	EMR	Self-study
		SME 16	Medication management, renewal, and symptom tracking	Patients and pharmacists	Private institutions (pharmacies)	Setup fee, subscription fee, pay per use	Views PHI (does not store or collect)	Local pharmacy server	Self-study
**Primary care**									
	**System services**								
		SME 19	Administrative optimization of clinics	Administrators, physicians, and patients	Public institutions (primary care clinics and specialist clinics) and private institutions (health and wellness)	Rental fee	Collects PHI	Patient reported	Self-study
		SME 20	Coordination of home visits	Patients, physicians, and caregivers	Physicians	Subscription fee, pay per use	Collects PHI	Patient reported	Self-study
**Home care**									
	**System services**								
		SME 21	Coordination of home care services	Patients and caregivers and personal support workers	Private institutions (home care)	Pay per use	Collects and transmits PHI	Patient reported	Self-study
**Physiotherapy**									
	**System services**								
		SME 22	Coordination of home visits	Patients	Patients and private insurance	Pay per use	Collects PHI	Patient reported	Self-study

^a^SME: small- and medium-sized enterprise.

^b^PHI: personal health information.

^c^EMR: electronic medical record.

^d^N/A: not applicable.

Two central themes were identified, each with 3 subthemes. First, a common barrier to system integration was the lack of formal evaluation, with SMEs having limited resources and opportunities to conduct such an evaluation. Second, the health system’s current structure does not create incentives for clinicians to use digital technologies, which threatens the sustainability of the SMEs’ business models.

### Lack of Access to Evaluation Resources Was a Barrier to Implementing Digital Technology in Clinical Settings

Digital technology SMEs lack access to clinical evaluations. This leads to uncertainty in the value these tools provide, which makes procurement by third parties highly challenging.

#### Lacking Evidence of Value

The advisors articulated several concerns regarding the lack of evidence on the value of the technology and lack of clarity on the issues that the technology sought to address. Fewer than half of the companies had conducted an external evaluation (Table 2). The advisors linked the absence of evidence on the effectiveness and value of downstream challenges with procurement and uptake. The advisors proposed that SMEs invest in generating robust clinical and economic evidence to substantiate claims—an important prerequisite for payers, providers, and other health care purchasers to identify the most impactful digital health products and services*:*

Generally, public sector payers [...] will be looking for clear evidence that a solution moves metrics along each side of what is known as the Triple Aim –improved health outcomes, improved experience of care, greater value (lower cost).

Excerpt from the SME 24 report

The advisors encouraged SMEs to identify meaningful metrics in early discussions with potential procurers to ensure alignment of evidence generated with information that supports procurement decisions. Evaluating a tool in a clinical environment provides evidence of value to clinicians, whose engagement is required to adopt the technology. The advisors cautioned that this evidence had to be curated from a trusted source with high methodological quality to ensure credibility:

The clinicians will immediately want to know the answers to the following before considering use of the device, and will want evidence to back up the answers: a. Is this device of equivalent diagnostic fidelity to other devices? b. Does having this information get me through my clinic more effectively? c. Does having this information allow me to make better decisions? d. Does this data, whether provided to clinician or patient, improve on a significant health outcome?

Excerpt from the SME 3 report

#### Methods for Generating Evidence

Recommended methods of evidence generation were often contingent on an SME’s stage of maturity and reach in the market. An SME’s decision not to conduct an external evaluation was typically related to a lack of funding and perceptions by SMEs that it was not a necessary precursor of market entry for their tool. The advisors frequently recommended that SMEs engage in small-scale external evaluations (ie, shorter duration and less cost) to meet system needs, including evidence of acceptability, usability, demand, integration, and implementation. SMEs at earlier stages of development were advised to run smaller pilot studies to refine the technology and business model as a prerequisite for scale to ensure user and payer value propositions were established:

[C]onduct a small-scale evaluation of [product] in an idealized population of practitioners and patients informed by the Triple Aim. A large-scale, multi-center evaluation will be costly and high risk of failure without smaller scale refinement. Ideally, this evaluation would occur with an experienced evaluation partner and the university setting may be ideal.

Excerpt from the SME 23 report

For more mature SMEs with existing, objective evidence of value, the advisors suggested a more comprehensive assessment of impact across the Institute for Health Improvement’s Triple Aim framework using a randomized control or pragmatic trial design. This was seen as a necessary step to establish external validity and generalizability, particularly before implementation within different contextual environments:

We recommend then running a trial to link your tool to outcomes for clinicians, patients, and the system (e.g. faster time to healing, reduced severity of wounds, reduction of complications, reduced cost, etc.).

Excerpt from the SME 5 report

#### Challenges in Executing Evaluations

It was widely acknowledged that the need for rapid advancement, evaluation, and distribution of digital health technologies was in direct conflict with the complex and conservative nature of health care research. Generating high-quality clinical evidence is expensive, cumbersome, and time consuming because of the administrative and regulatory requirements imposed by health systems for testing in real-world clinical environments. This challenge is further exacerbated by the fact that measuring outcomes postmarket entry (eg, impact on patient health outcomes and health system costs) often requires high-resource, large-scale studies. For certain SMEs, generating evidence was further derailed because their service was designed to address factors upstream of patient care (eg, health promotion or educational initiatives), making direct measurement of their impact difficult:

Determine your impact on health institutions and systems: Before you move into the clinical space, it is important to understand the value proposition brought forth by [product]. The place where this application will offer value (e.g. in system cost-savings, improved patient care, in the patient’s home) will determine who to target as a customer.

Excerpt from the SME 12 report

The advisors provided 2 main solutions to address SME barriers. First, several funding sources, namely, grant opportunities, were identified to highlight opportunities for SMEs to access financial resources to conduct an evaluative study. Second, the advisors suggested strategic partnerships with clinical organizations and health system experts who could help them navigate the complex clinical and institutional requirements to run a high-quality evaluation and to access clinical environments to run a trial:

Forging relationships is critical to your success. It is important that you [...] have dedicated resources in this task. Also recognize that international experience is nice, but what you truly need is experience working with the depth and breadth of entities in the Canadian context if you are to be successful in Canada.

Excerpt from the SME 18 report

### The Sustainability of Business Models Was Often Threatened by Inefficiencies in the Current System Structures, Which Minimize Incentives to Use Digital Technologies

System-level incentives for care provisions are outdated and discourage clinicians from adopting digital technology and misalign drivers of institutional procurement and user adoption. The 3 primary structural barriers repeatedly identified throughout the reports were the lack of (1) physician incentives for use of digital technologies, (2) public funding for hospitals and clinics to use digital tools, and (3) incentives to use digital tools for care coordination.

#### Creating Incentives for Physicians as Users

The viability of clinicians as primary users of a technology is often highly contingent on, or at times in conflict with, their prevailing funding model. For example, in Ontario, during this study, when physicians engaged with a patient virtually *in lieu of* seeing a patient in-person, there was a significant reduction in revenue for similar, or at times greater, clinical effort (eg with tools whose clinical case is predicated on real-time data monitoring). There is no remuneration model for data monitoring outside the boundaries of an office visit, which is at odds with the use case proposed by some SMEs:

Consider the clinical integration. If [product] is integrated in the ways the business plan suggests (i.e. as a “billable” / “prescribe-able” [product] in the Canadian context), there will need to be real consideration of the existing clinical workflows; and how [product] will fit into them. This becomes most important for integration for whichever clinical care provider acts as the “recipient” of data or notifications from the platform. This is further complicated when considering multiple disease specific platforms for management. Recognize that, unfortunately, financial incentives are not available to clinicians to manage this type of data–many who would be receiving them are fee for service.

Excerpt from the SME 10 report

The advisors recommended that SMEs could address this by better understanding the funding models in which their technologies fit and attempting to construct business cases around them. For example, in a fee-for-service system, there is value in a technology that increases throughput relative to the necessary increase in effort. In the case of SME 6, the proposed technology enables patients to monitor their wound healing at home and improve clinical triage by leveraging smartphones to take images over time. Reviewing these data with the patient has the potential to reduce the amount of time required for in-person appointments by allowing a rapid review of images. This is in contrast to the current standard of obtaining a complex history using subjective wound and skin descriptors that are challenging for patients to understand. Relatedly, the advisors recommended that SMEs leave physicians out of the equation, as involving busy clinicians unnecessarily creates obstacles to the business case. Involving physicians often involves significant behavioral and clinical workflow changes, which can be difficult to convince them to do without dedicated funding:

The current workflow needs to consider change in practice. If a clinician administers surveys, it is often through paper and pencil. The use of digital technology could improve this. However, a significant barrier to scale is that most clinicians do not use ongoing monitoring in their practice and would require significant behaviour change and clinical education.

Excerpt from the SME 7 report

#### Public Versus Private Payers

Most SMEs proposed several potential payers for their technology, which makes it difficult to establish a sustainable business model. There were common challenges in identifying value propositions for publicly funded hospitals and clinics to procure digital technologies because hospitals are funded through the provision of in-person services:

Selling to hospitals is an unlikely market: The value proposition for healthcare institutions such as hospitals is unclear, as there is no reimbursement for real time remote monitoring (only retrospective). Further, you would have to go through burdensome procurement processes to be used.

Excerpt from the SME 2 report

Many SMEs proposed that the value-add for organizations was reducing the number of clinical visits. However, innovators failed to recognize that system benefits (eg, overall reduction in system use because of improved patient outcomes) are not often reflected at the institutional level. Institutions have minimal incentive to procure a technology that fixes on a *system* issue but takes away from their own business and revenue:

Market can be challenging in that there are system-level incentives but health institutions see minimal benefit: There may be difficulty in obtaining buy-in since benefits/cost savings accrue at the system-level, but the institutions could actually lose money if, for example, this were used to reduce the number of visits by a homecare agency or to a wound care clinic.

Excerpt from the SME 5 report

The advisors frequently recommended that the SMEs sell to private clinics as an alternative to public payers, as there are more direct incentives to procure technologies that reduce the need for in-person visits or time spent with salaried clinicians:

Consider marketing this to institutions with competitive markets: This could include ancillary services such as physiotherapy, chiropody, chiropractic, ultrasound clinics, etc., that need to attract customers. They could use patient feedback to improve their services and market their satisfaction rates.

Excerpt from the SME 25 report

Given that direct reimbursement for many digital technologies (such as remote monitoring of patient data) is not available at scale across the system, the advisors recommended that companies consider partnerships with insurance companies or employee assistance plans, whereby digital technologies can help identify and effectively manage *at-risk* groups. In Canada, private insurance companies that are funded primarily through employer benefits programs are increasingly paying for supplemental digital health services such as virtual medical appointments, health tracking tools, and other benefits that reduce missed days at work.

#### Creating Incentives for Care Coordination Between Fragmented Systems

Most technologies boasted the functionality of collecting and storing personal health information, which led SMEs to construct a value proposition around sharing this information across clinician groups to improve decision making. Unfortunately, the siloed reality of the health system meant that there were rarely preexisting channels for data and information sharing, undermining the ability for the technology to realize its stated value across institutional boundaries:

In homecare, there are multiple providers involved in the care of patients. It is important to understand how this information can be shared between them and who is responsible for acting upon the information. EMR integration or EMR-compatible files can be valuable in sharing information.

Excerpt from the SME 6 report

In the absence of preexisting practices for effective data sharing, SMEs are tasked not only with providing the mechanism for sharing this information but also with creating a model that creates incentives for institutions and clinicians to change their behavior to send and receive these data:

Your overview also talks about integrating systems that currently don’t integrate (i.e. pharmacies). This is not a technical problem but is largely a bureaucratic and political problem. What role will you play in sorting those issues out (if any)?

Excerpt from the SME 1 report

Technology interoperability and integration were central issues. Most clinical organizations communicated to SMEs that they wanted everything to integrate into their electronic medical record (EMR) to minimize workflow disruption. However, it becomes highly burdensome for SMEs to seamlessly integrate into EMRs across health institutions because most institutions have distinct, noninteroperable EMRs. As such, SMEs carry the burden of reconfiguring their technology for the unique integration needs of each health institution, creating an obstacle for scalability and broader system transformation.

## Discussion

### Principal Findings

The 25 case studies presented in this study exemplify the challenges of developing a successful, evidence-informed business model for digital health technologies in publicly funded systems. Consistently, these SMEs faced significant challenges in engaging users and payers from the public system due to perverse economic incentives. Physicians are compensated by in-person visits, which actively works against the goals of many digital health technologies to keep patients out of clinics and hospitals. Many hospital payment models are based on visit volumes, where there are no incentives to invest in technologies that improve patient and system outcomes and, in turn, reduce volumes. Creating de novo fee codes from a government for uncovered virtual care services is complex, bureaucratic, time consuming, and infeasible for an SME to pursue. Further, reluctance from health systems, institutions, and providers to engage with digital health often relates to deficient evidence of clinical value. Funding for the evaluation of such innovations, including resources for proper implementation, which is costly for resource-constrained recipient sites, is lacking in public health systems.

COVID-19 has introduced rapid shifts in funding models and encouraged the adoption of digital technologies due to the high cost of physical contact. However, it is unclear what billing changes will be sustained, and innovative technology is yet to be adopted at scale. Although there is mass adoption of virtual visits, incentives for clinicians in publicly funded systems to use innovative care models such as remote monitoring and clinical decision support remain unclear.

### Comparisons With Prior Work

This paper adds to the existing literature by providing a report and analysis of health system barriers faced by real SMEs developing digital health technologies for a publicly funded system. The existing literature consists mostly of policy analysis, wherein academics propose models to improve the evaluation and implementation of digital technologies without case studies of actual SMEs. We could find no comparative literature that assesses existing SMEs’ business and clinical models in a public health system. This paper, therefore, grounds many of the policy findings produced in previous literature in real-world examples.

The barriers identified are not unique to the Canadian health system. Globally, the potential of digital health has scarcely been realized, partly due to SMEs’ difficulties in generating rapid and robust evidence to guide investment decisions [3,16,22]. There is tension between industry and health system actors—digital technology evolves rapidly, whereas research is slow and arduous, often taking years to determine that an intervention leads to effective outcomes [3,23]. Most health professionals seek randomized controlled trials as proof of value [24,25]. However, to minimize the risk of failure, SMEs with less mature technologies would benefit from smaller evaluations to refine their products before engaging in large trials. Due to complex implementation and contextual factors (eg, user experience, engagement, and effectiveness) that affect digital health evaluation, and evaluation timelines that are at odds with the modern ultra-rapid evolution of digital tools, there is a need to reduce our reliance on some traditional approaches to generating evidence, such as randomized controlled trials [26].

Conducting a rigorous evaluation is expensive and time consuming, and public funding for digital health research is minimal compared with funding for biomedical research [23]. Further barriers include acquiring clinical expertise, accessing clinical environments for iterative testing, and navigating complex regulatory and ethical standards [22,26,27]. Although there have been some efforts to standardize approaches to digital health evaluations [16,22,27,28], few jurisdictions have attempted to minimize barriers to evidence generation. One promising avenue to address these barriers is investment in publicly funded programs that support collaborative studies between researchers, health care organizations, and SMEs. For instance, Health Innovation Manchester offers grant opportunities to market-ready SMEs demonstrating strong evidence of improving population health outcomes, which can be used to support further evaluation activities [29]. There are also several programs and accelerators that offer advice to small businesses on navigating health systems to identify a sustainable business model, including the program outlined in this paper [27,29-31]. Academic medical centers have played a role in advancing digital health in the United States with programs to connect innovators to funding, evaluation, clinical expertise, and business development support [23]. In Ontario, some of our client companies were able to benefit from the Health Technologies Fund implemented in 2016, with the goal of increasing investment in, use of, and evaluation of digital tools in practice. This grant provided dedicated funding for clinical organizations, SMEs, and researchers to implement and evaluate digital health tools in clinical settings. However, it was ultimately canceled [32].

All SMEs who went through the consulting program self-reported having identified issues in the health system themselves and then having developed digital technologies to solve those problems. Many SMEs consulted health system partners (primarily clinicians) in developing their technologies; however, some developed their technologies in isolation. Isolated development required SMEs to convince clinicians and organizations to procure and use their technologies *off the shelf*, which required significantly more effort to achieve clinical buy-in. One method to address the incentive barrier would be for health organizations to identify problems and to match SMEs to collaboratively solve those problems alongside end users. This would ensure that health organizations and providers are encouraged to use the technology [33]. However, funding to support implementation and sustained use remains a barrier, and scalability can be a challenge if technologies are so locally tailored that they are not generalizable within a broader context.

### Limitations

As this paper reports on a real-world consulting program, the results are more limited than those of a typical research study. We were limited by the fact that the information provided by the SMEs and the analysis conducted by the advisors was based on a single interaction that spanned approximately 1 month. As such, we did not have longitudinal data on the challenges faced by the SMEs and the future success of the companies after their engagement with the program. Further, data on barriers were not retrieved by directed interviews but rather by coding key barriers identified by an expert committee engaged in a pragmatic consulting process with real companies. The barriers reported are those that emerged naturally in the consultations with those SMEs, rather than through a targeted research methodology.

### Conclusions

In 2006, Herzlinger [34] wrote about why innovation in health care is so challenging, citing conflicting motivations between stakeholders, lack of funding for development and sustainability, and demanding accountability from technology developers as key barriers. In 2020, these barriers persist. COVID-19 has introduced an unprecedented need to provide care through virtual means. To promote high-quality care remotely, innovative digital technologies will be essential. To encourage their adoption, the system must prioritize evaluation and the creation of incentive models that support uptake or risk ongoing stifling of promising innovations. Specific tensions exist between the economic incentives of clinical organizations and the use of tools that would benefit patients’ well-being, ultimately because of a disconnect of the primary beneficiaries of tools and those who pay for and use them. This means that SMEs have a greater opportunity for success, growth, scale, and sustainability in private markets. There is a need in publicly funded health systems for dedicated funding for the evaluation of digital technologies, streamlined pathways to clinical integration, and system entry points through aligned incentives of users and payers to improve patient outcomes.

## Appendix

Multimedia Appendix 1 The Women’s College Hospital Institute for Health System Solutions and Virtual Care digital technology project on-boarding form.

## Abbreviations

CHA: Canada Health Act
EMR: electronic medical record
SME: small- and medium-sized enterprise
WIHV: Women’s College Hospital Institute for Health System Solutions and Virtual Care
