# Supplementary material for: Barriers to Creating Scalable Business Models for Digital Health Innovation in Public Systems: Qualitative Case Study
Source: JMIR Public Health Surveill. 2020 Dec 10;6(4):e20579. doi: 10.2196/20579 (PMC7759439; doi:10.2196/20579)
Supplement: Multimedia Appendix 1 [file publichealth_v6i4e20579_app1.docx]

## Appendix

**WIHV Digital Technology Project On-Boarding Form**

Profile Questions

- Name of company, product, or service for consideration by WIHV
- Primary Contact Information
- Is your firm an incorporated, for-profit company?
- Is it a small or medium-sized enterprise (SME) with less than 500 employees?
- Is the company operating in Canada?
- Is there an objective to grow and generate profits?
- Is there at least one non-owner on payroll?
- Year Started
- Funding Stage (e.g. seed, series A, series B, publicly traded, etc.)
- Does the company/product/service generate revenue? If yes, what types of clients do you currently have?

Purpose of Submission

- Please articulate your reason for submitting this on-boarding form. What questions are you hoping to have answered? What outcomes are you seeking?

Product Demonstration

- Please include a link to a video demonstration or attach a presentation which overviews your product.

Business Partners and Team Members

- Please list all business partners and team members who will be involved in this project. Include the name, roll and email of each individual.

Overview of the company

- Please provide a brief overview of you company. Please include a description of the product, service, and unique value proposition that you provide.
- Specifically, how will your product affect patients?
- Specifically, how will your product impact healthcare providers?
- Specifically, how will your product impact institutions (pharmacies, homecare, hospitals, etc)?
- Specifically, how will your product impact the system (efficiency, costs, access, quality, etc)?
- Why do you think your idea will work? Has anything similar been tried anywhere before? Are there scientific evaluations that you know of?
- Why are you passionate about your company? What unique skills/experiences do you and your team members bring to the company?

Your Team

- What skills/experiences do you feel you are lacking?

Your Users

- Who are your target users?
- What is your design/engineering process? (How do your ideas become a fully functional product?)
- Have any users interacted with your products? What feedback did you receive and what did you learn? What is your approach to seeking user feedback?

Your Customers

- Who are your target customers? Who are your target users? Who will pay for it and why? What is your potential market? (How many potential customers, etc.)?
- Have you thought about how much your customers would pay? How often? How? (Directly, indirectly through insurance, etc.)
- What is your strategy for reaching your customers/scaling your product? What are the major things holding you back?

Other Questions

- Please include a basic description of the product(s) and the intellectual property assets (without providing confidential information).
- Does your product capture, store or transmit personal health information (PHI)? Describe your approach to patient privacy and data security.
- Do you know of any regulations/certifications that are relevant to your product (e.g. Health Canada, International Standards Organization, etc.)? Have you sought regulatory approval? How does your product comply with those approvals?
- How did you hear about this program?
- Please provide any additional comments you may have here.
